# Meaningful human control and variable autonomy in human-robot teams for firefighting

**DOI:** 10.3389/frobt.2024.1323980

**Published:** 2024-02-01

**Authors:** Ruben S. Verhagen, Mark A. Neerincx, Myrthe L. Tielman

**Affiliations:** ^1^ Interactive Intelligence, Intelligent Systems Department, Delft University of Technology, Delft, Netherlands; ^2^ AI*MAN Lab, Intelligent Systems Department, Delft University of Technology, Delft, Netherlands; ^3^ Human-Machine Teaming, Netherlands Organization for Applied Scientific Research (TNO), Soesterberg, Netherlands

**Keywords:** meaningful human control, variable autonomy, human-robot teams, dynamic task allocation, thematic analysis

## Abstract

**Introduction:** Humans and robots are increasingly collaborating on complex tasks such as firefighting. As robots are becoming more autonomous, collaboration in human-robot teams should be combined with meaningful human control. Variable autonomy approaches can ensure meaningful human control over robots by satisfying accountability, responsibility, and transparency. To verify whether variable autonomy approaches truly ensure meaningful human control, the concept should be operationalized to allow its measurement. So far, designers of variable autonomy approaches lack metrics to systematically address meaningful human control.

**Methods:** Therefore, this qualitative focus group (*n* = 5 experts) explored quantitative operationalizations of meaningful human control during dynamic task allocation using variable autonomy in human-robot teams for firefighting. This variable autonomy approach requires dynamic allocation of moral decisions to humans and non-moral decisions to robots, using robot identification of moral sensitivity. We analyzed the data of the focus group using reflexive thematic analysis.

**Results:** Results highlight the usefulness of quantifying the traceability requirement of meaningful human control, and how situation awareness and performance can be used to objectively measure aspects of the traceability requirement. Moreover, results emphasize that team and robot outcomes can be used to verify meaningful human control but that identifying reasons underlying these outcomes determines the level of meaningful human control.

**Discussion:** Based on our results, we propose an evaluation method that can verify if dynamic task allocation using variable autonomy in human-robot teams for firefighting ensures meaningful human control over the robot. This method involves subjectively and objectively quantifying traceability using human responses during and after simulations of the collaboration. In addition, the method involves semi-structured interviews after the simulation to identify reasons underlying outcomes and suggestions to improve the variable autonomy approach.

## 1 Introduction

Humans and robots are increasingly working together in human-robot teams on complex tasks ranging from medical surgery to firefighting. For example, the fire department of Rotterdam in the Netherlands is already using explore and extinguish robots for situations too dangerous for firefighters. Several factors determine the success of these human-robot teams, such as situation awareness, mutual trust, and common ground (Klein et al., 2004; Salas et al., 2005). The ultimate goal of human-robot teams is harnessing the combination of strengths of both humans and robots, to accomplish what neither can do alone (Akata et al., 2020). Such an integration of robots that augment rather than replace humans requires robots to dynamically vary their level of autonomy to collaborate with humans efficiently.

Rapid developments in the field of robotics and artificial intelligence allow robots to become increasingly autonomous and perform tasks without much human intervention and control (Santoni de Sio and Van den Hoven, 2018). However, since robots do not have a legal position, humans should be held accountable in case robot behavior does not comply with moral or ethical guidelines (van der Waa et al., 2021). Therefore, higher levels of robot autonomy should be combined with meaningful human control and human moral responsibility (Santoni de Sio and Van den Hoven, 2018; Santoni de Sio and Mecacci, 2021). The concept of meaningful human control is based on the assumption that human persons and institutions should ultimately remain in control of, and thus morally responsible for, the behaviour of intelligent autonomous systems like robots (Santoni de Sio and Van den Hoven, 2018). Meaningful human control originated from the discussion on autonomous weapon systems but its relevance quickly expanded to intelligent (semi)autonomous systems in general.

One of the first works on meaningful human control was a philosophical account towards two necessary conditions: the tracking and tracing conditions. In short, the tracing condition implies that a system’s behaviour, capabilities, and possible effects should be traceable to a proper moral and technical understanding of at least one relevant human agent who designs or interacts with the system. On the other hand, the tracking condition implies that a system should be responsive to the human moral reasons relevant to specific circumstances (Santoni de Sio and Van den Hoven, 2018). Designing for meaningful human control means designing for human moral responsibility and ensuring humans are aware and equipped to act upon their moral responsibility. By doing so, responsibility gaps in culpability, moral and public accountability, and active responsibility can be avoided (Douer and Meyer, 2020; Santoni de Sio and Mecacci, 2021; Cavalcante Siebert et al., 2022; Veluwenkamp, 2022). Several solutions for addressing meaningful human control in human-robot teams have been proposed, such as team design patterns (van Diggelen and Johnson, 2019), value sensitive design (Friedman and Hendry, 2019), machine ethics (Anderson and Anderson, 2007), and variable autonomy (Methnani et al., 2021).

Variable autonomy refers to the ability to dynamically adjust the levels of autonomy of a system, for example, by switching the level of autonomy from full robot autonomy to complete human operator control (Chiou et al., 2021). Variable autonomy is often used to describe human-robot teams in which the level of robot autonomy varies depending on the context. One of the main goals of variable autonomy approaches is to maximise human control without burdening the human operator with an unmanageable amount of detailed operational decisions (Wolf et al., 2013; Chiou et al., 2016). For example, in human-robot teams for firefighting variable autonomy can be used to dynamically allocate moral decision-making to humans and non-moral decision-making to robots. It is argued that robots with variable autonomy can ensure meaningful human control over these robots by satisfying accountability, responsibility, and transparency (Methnani et al., 2021). However, testing whether variable autonomy approaches truly ensure meaningful human control is crucial before actually adopting them. Unfortunately, designers of variable autonomy approaches lack metrics needed for systematically addressing meaningful human control (Canellas and Haga, 2015; Douer and Meyer, 2020). On the other hand, meaningful human control is already increasingly being imposed as a requirement for variable autonomy approaches.

Imposing meaningful human control as a requirement and verifying if variable autonomy approaches indeed fulfill this requirement means we must be able to measure meaningful human control (van der Waa et al., 2021). Therefore, turning the abstract concept of meaningful human control into measurable observations (i.e., operationalize) is required. So far, only a few approaches for operationalizing meaningful human control exist (Calvert et al., 2020; van der Waa et al., 2021). Therefore, this qualitative study explores different approaches to measure meaningful human control during dynamic task allocation using variable autonomy in human-robot teams for firefighting, aimed at creating an evaluation method. We will first discuss the context and variable autonomy approach in more detail, as well as existing operationalizations of meaningful human control (Section 2). Next, we will discuss how we conducted our study (Section 3), followed by the results (Section 4). Finally, we will present a discussion, propose an evaluation method of meaningful human control, and conclude our work (Section 5).

## 2 Background

### 2.1 Moral decisions in human-robot teams for firefighting

Explore and extinguish robots are increasingly collaborating with firefighters to detect victims and extinguish fires in properties too dangerous for firefighters, for example, because the structural condition is unsafe. Currently, firefighting robots are mostly teleoperated by firefighters, allowing an otherwise impossible offensive inside deployment aimed at fighting the fire and rescuing people. These firefighting robots are equipped with several cameras (thermal imaging, RGB, pinhole), sensors (LIDAR, temperature, explosion danger), and capabilities (water shield protection, fire hose), enabling navigation, localization, detection, protection, mapping, and extinguishing. The information provided by the robot’s sensors is crucial for firefighters to make decisions about localizing the fire source, rescuing victims, switching deployment tactic, extinguishing or evacuating, and sending in firefighters to help. The collaboration between firefighters and their firefighting robot demonstrates how human-robot teams can harness the combination of strengths of both parties, to accomplish what neither could do alone.

Although these teleoperated firefighting robots are already of great use, there is a strong preference within the field of rescue robotics for (semi)autonomous robot behavior to reduce the workload of the operator (Delmerico et al., 2019). The potential of artificial intelligence provides great opportunities for making these robots more autonomous, and some progress has already been made (Kruijff et al., 2014; Kruijff-Korbayová et al., 2015; Frering et al., 2022). It is considered important, however, to actively keep a human involved in the collaboration to guide the robot’s behavior during the mission (Delmerico et al., 2019). Variable autonomy will be crucial to effectively implement this collaboration between a human operator and increasingly autonomous firefighting robot because it can increase human control while decreasing the workload of the human operator (Wolf et al., 2013; Chiou et al., 2016).

This increase in robot autonomy raises important challenges such as how to design for meaningful human control in these human-robot teams. Designing for meaningful human control is crucial in human-robot teams for firefighting because the scenario involves morally sensitive situations (i.e., situations in which something one might do or is doing can affect the welfare, rights, and values of someone else either directly or indirectly (Rest, 1994)). These morally sensitive situations can involve deciding to preserve the safety of firefighters if that means the life of victims cannot be rescued. If the robot would autonomously make an incorrect moral decision in such situations, consequences could be the loss of lives and responsibility gaps (Santoni de Sio and Mecacci, 2021). Therefore, especially when human-robot teams are tasked with making moral decisions, meaningful human control is crucial to ensure humans can be held accountable for robot behavior (van der Waa et al., 2021).

Team designs patterns have been applied to describe the allocation of tasks for moral decision-making in human-robot teams (van der Waa et al., 2020; van Diggelen et al., 2023). These patterns can express forms of collaboration with various team properties by task-independently describing 1) how humans and robots collaborate and communicate; 2) the requirements needed to do so; and 3) advantages and disadvantages when being applied (Van Diggelen et al., 2018; van Diggelen and Johnson, 2019). Various team design patterns have been constructed to address moral decision-making in human-robot teams, often manipulating the level of human and robotic moral agency. For example, supported moral decision-making requires human moral supervision over a robot and taking over when perceiving the need for moral decisions. The robot should then support the human during moral decision-making by explaining the moral context. Another example is fully autonomous moral decision-making. In this collaboration design, human values are implemented in the robot, allowing it to autonomously make moral decisions. If these artificial agents would make moral decisions violating ethical guidelines and moral values, the tracking and tracing conditions should allow the identification of responsible humans to hold accountable (Santoni de Sio and Van den Hoven, 2018). However, we are not convinced that fully autonomous artificial moral agents are feasible and desirable during collaboration with humans. Instead, we believe that variable autonomy can be used to dynamically allocate all moral decisions to humans and non-moral decisions to robots.

### 2.2 Dynamic task allocation using variable autonomy

In robots with variable autonomy, humans can take control over certain (or all) elements of robot behavior (Methnani et al., 2021). A common distinction in human oversight and control over robots with variable autonomy involves three levels: having humans-in-the-loop, humans-off-the-loop, or humans-on-the-loop (Crootof, 2016; Methnani et al., 2021). Maintaining humans-in-the-loop requires informed human approval for all elements of robot behavior, for example, during complete tele-operation of firefighting robots. In contrast, allowing humans-off-the-loop refers to fully autonomous robots without human operator involvement, for example, firefighting robots that autonomously explore burning buildings and make moral decisions. Finally, having humans-on-the-loop assumes a supervisory human role tasked with monitoring and influencing robot behavior when necessary, for example, when firefighters overrule the trajectory of firefighting robots or intervene when perceiving the need for moral decisions.

Another example of having humans-on-the-loop during moral decision-making in human-robot teams for firefighting is dynamic task allocation using variable autonomy (Table 1). In this variable autonomy approach, human moral values are elicited and implemented in the robot, allowing robot identification of morally sensitive situations. Eliciting human values for implementing artificial moral agents is a complex and multifaceted process, and there is a lot of discussion on its need and feasibility. Nevertheless, there are several approaches for value elicitation, each with its own strengths and weaknesses. For example, a rule-based elicitation questionnaire can be used where participant responses directly influence an autonomous agent’s behavior through predefined rules (van der Waa et al., 2021). Another example is using advanced machine learning and natural language processing techniques to infer and reason about human moral values (Jiang et al., 2021; Liscio et al., 2021). For dynamic allocation of moral decisions during firefighting, a questionnaire and crowdsourcing approach could be suitable to identify and use moral features as predictors of moral sensitivity (e.g., the number of victims, fire duration, and risk of building collapse).

**TABLE 1:** Variable autonomy approach for human-robot teams engaged in moral decision-making, where tasks are allocated dynamically (slight adaptation of the team design pattern by van der Waa et al. (2021)). The variable autonomy approach is communicated in the form of a team design pattern that describes the collaboration, structure, requirements, advantages, and disadvantages of the approach.

Name	Dynamic task allocation using variable autonomy
Description	Human moral values are elicited and implemented in the robot, allowing the robot to identify morally sensitive situations. When the robot classifies situations as morally sensitive, it allocates the related tasks/decisions to the human operator, while taking on the rest itself. The human operator can alter this allocation and intervene at any time. The robot explains allocations, non-moral decisions, and the moral context
Structure	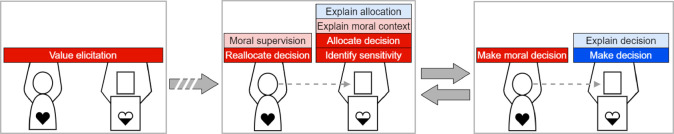
Requirements	**R1** The robot should be sufficiently able to identify morally sensitive situations
	**R2** Robot explanations should raise human moral awareness during supervision
	**R3** Robot explanations should not bias the human operator in its decision-making
Advantages	**A1** The robot reduces the workload of the human operator
	**A2** The human operator is in control of all morally sensitive decisions
	**A3** Robot explanations can build appropriate mental models of the robot
Disadvantages	**D1** The human operator does not make all decisions
	**D2** Interpreting robot explanations and allocations requires time
	**D3** Operator under-/overload can result in missed moral decisions made by the robot

After this value elicitation process, the firefighting robot should autonomously perform its explore and extinguish tasks while being morally supervised by the human operator who retains the power to override the robot’s behaviour (Abbink et al., 2018; van der Waa et al., 2020). Using variable autonomy, the robot identifies morally sensitive situations and allocates moral decision-making in these situations to the human operator, while making all non-moral decisions itself. This way, the variable autonomy approach ensures that humans can be held accountable for moral decisions and robot behavior, while the robot can decrease the workload of firefighters by preventing them from exercising control unnecessary often.

Variable autonomy approaches vary in terms of which aspects of autonomy are adjusted, by whom, how, why, and when (Castelfranchi and Falcone, 2003; Bradshaw et al., 2004; Methnani et al., 2021). The variable autonomy approach in Table 1 adjusts robot decision-making, and these adjustments are executed by either the human operator or robot (i.e., a mixed-initiative approach). The robot is primarily responsible for autonomy adjustments when it identifies situations as morally sensitive and requiring human moral decision-making. In contrast, the human is responsible for autonomy adjustments when during moral supervision he/she intervenes when the robot attempts to make moral decisions because it incorrectly identified moral sensitivity. During dynamic task allocation using variable autonomy, the autonomy level is adjusted in a discrete way from (semi-)autonomous robot decision-making in not morally sensitive situations to manual human decision-making in morally sensitive situations. The reasons for adjusting autonomy to complete human control in morally sensitive situations are pre-emptive to ensure meaningful human control. Finally, autonomy adjustments are executed during active operation of the robot in real firefighting scenarios by responding to changes in the moral sensitivity of situations.

For variable autonomy approaches to be effective, it is important to explicitly define which entities (human, robot, or both) are capable and responsible for which tasks (Methnani et al., 2021). Using team design patterns to describe the variable autonomy approach provides such a definition of roles and responsibilities and determines who transfers control of what, when and why it is needed, and to whom. To ensure adequate fulfillment of defined roles and responsibilities, there must be an appropriate means for information exchange allowing the states of the robot and environment to be understood. Moreover, this means of information exchange should achieve situation awareness and appropriate trust calibration without overloading the human operator’s cognitive abilities (Endsley, 1988; Lee and See, 2004; Methnani et al., 2021). Therefore, robot explanations are crucial during dynamic task allocation using variable autonomy in human-robot teams for firefighting. More specifically, the robot should involve and support the human operator by explaining the moral context and its non-moral and allocation decisions. It is crucial that these robot explanations do not 1) bias the human operator in its decision-making; 2) reduce situation awareness by information overload; or 3) cause misuse or disuse by information underload (Lee and See, 2004; van der Waa et al., 2021). However, without these robot explanations the human operator will not be able to exercise control in a timely and accurate manner (van der Waa et al., 2021).

The explanations of the robot are especially important when it classifies situations as not morally sensitive and allocates decision-making to itself. We suggest that the responses of the human operator when the robot allocates decision-making to itself can be can be classified using signal detection theory (Wickens, 2001; Douer and Meyer, 2020). More specifically, this classification considers the presence or absence of human reallocation interventions, robot classification of situations as morally sensitive or not, and the true nature of situations as morally sensitive or not. For example, hits refer to human reallocation interventions when the robot classifies morally sensitive situations as not morally sensitive. In contrast, misses refer to no human interventions when the robot classifies morally sensitive situations as not morally sensitive. On the other hand, false alarms refer to human reallocation interventions when the robot classifies not morally sensitive situations as not morally sensitive. Finally, correct rejections refer to no human interventions when the robot classifies not morally sensitive situations as not morally sensitive. From a meaningful human control perspective, one could argue that hits and misses are crucial to ensure human moral decision-making, whereas false alarms and correct rejections are less problematic. Classifying human operator responses using signal detection theory provides quantitative measures that can be applied to verify if the variable autonomy approach truly ensures meaningful human control.

### 2.3 Existing operationalizations of meaningful human control

Imposing meaningful human control as a requirement and verifying if variable autonomy approaches indeed fulfill this requirement calls for methods to measure meaningful human control. So far, only few operationalizations of meaningful human control have been proposed. One of them introduced three measurable dimensions of meaningful human control: 1) Experienced meaningful human control and behavioral compliance with 2) ethical guidelines and 3) moral values (van der Waa et al., 2021). The authors argue that humans experience meaningful human control, which can be measured subjectively. Moreover, they argue that the behavioral compliance with moral values and ethical guidelines provides evidence for meaningful human control. In their work, they measure experienced control with a semi-structured interview using eight five-point Likert scale statements on concepts like time pressure, responsibility, and decision-making comfort and quality.

Another operationalization are the four necessary properties for human-robot teams to be under meaningful human control (Cavalcante Siebert et al., 2022). The first property requires an explicitly specified moral operational design domain where the robot should adhere to. This involves norms and values to be considered and respected during design and operation. Here, it is important that the robot embeds concrete solutions to constrain actions of the team within the boundaries of the moral operational design domain. Moreover, it is crucial that humans are aware of their responsibilities to make conscious decisions if and when the team deviates from the boundaries of the moral operational design domain. The second property requires humans and robots to have appropriate and mutually compatible representations of each other and the team, to decide which actions to take and perform. These representations should include reasons, tasks, desired outcomes, role distributions, preferences, capabilities, and limitations. Building these mental models of both team members can be achieved by for example, communication and explanations. The third property requires relevant human agents to have the ability and authority to control the robot, so that they can act upon their moral responsibility. This means humans should be able to change the robot’s goals and behavior to track reasons, as well as intervene and correct robot behavior. Here, it is important to clearly and consistently define role distributions, task allocations, and control authority. Again, team design patterns are particularly useful for describing and communicating such design choices. Finally, the fourth property requires the actions of the robot to be explicitly linked to actions of humans who are aware of their moral responsibility. This means the human-robot team should simplify and aid achieving human moral awareness, for example, using explanations of the robot’s actions.

In contrast to operationalizing the whole concept of meaningful human control, other studies operationalized only its tracing condition. For example, the cascade evaluation approach subjectively quantifies traceability (Calvert et al., 2020; Calvert and Mecacci, 2020; de Sio et al., 2022). This approach is centered around four aspects: 1) The exertion of operational control; 2) the involvement of a human agent; 3) the ability of a human agent to understand and interact with a robot; and 4) the ability of a human agent to understand their moral responsibility over a robot. For each aspect, involved human agents (e.g., operator or designer) are given a score along a six-point Likert scale, reflecting the degree of that aspect for the human agent. However, each aspect is considered as part of overall traceability, and therefore the scores from the previous and current aspects are compared to determine critical scores. This way, the critical scores for aspects 2, 3, and 4 are all influenced by the aspects that preceded them. Ultimately, the critical score of aspect 4 reflects the final traceability score. This operationalization of traceability is suitable for both a-priori and a-posteriori evaluation of robots and/or variable autonomy approaches. For example, the authors apply the cascade approach by presuming the situation of an inattentive driver struggling with the ability to retake control of an automated vehicle. However, the approach can also be applied to a-posteriori evaluate a variable autonomy approach by involved human agents or a third party.

The tracking condition of meaningful human control has been operationalized as reason-responsiveness (i.e., robots being responsive to human reasons to act) (Mecacci and Santoni de Sio, 2020). Here, the main idea is that the humans whose reasons are being tracked have the kind of control over robots that make them morally responsible for the actions of robots. A proximity scale has been introduced to identify and order human reasons according to proximity and complexity with respect to how closely they influence robot behavior (Mecacci and Santoni de Sio, 2020). In decreasing order of proximity and complexity, a distinction is made between the reasons values, norms, plans, and intentions. The authors argue that more proximal reasons (e.g., the intention to reallocate decision-making) are often closer in time to robot behavior and also simpler than more distal reasons (e.g., the plan to rescue all victims). However, this operationalization of tracking as reason-responsiveness has been questioned by demonstrating it is ambiguous in distinguishing between motivating and normative reasons (Veluwenkamp, 2022). More specifically, it is argued that tracking is operationalized in terms of motivating reasons (mental states) instead of normative reasons (facts), while the idea of responsibility attribution is derived from normative reason-responsiveness. Furthermore, this work shows that tracking cannot play an important role in responsibility attribution because normative reasons are agent-neutral (i.e., a fact for agent A is also a fact for agent B). Therefore, the author proposes that the tracing condition should be the sole determinant of responsibility, and that the humans to which robot actions can be traced back are the humans in control of and responsible for robot outcomes. Consequently, one could argue that verifying if dynamic task allocation using variable autonomy indeed ensures meaningful human control only requires measuring traceability.

These discussed operationalizations of meaningful human control demonstrate that only a few properties are actually transformed into quantifiable measures, while most properties still remain hard to quantify. Furthermore, the quantitative measures are all subjective such as experienced control (van der Waa et al., 2021) or the subjective traceability score (Calvert et al., 2020; Calvert and Mecacci, 2020; de Sio et al., 2022). To impose meaningful human control as a requirement and verify if variable autonomy approaches fulfill this requirement, more quantitative operationalizations and objective measures are appreciated.

## 3 Methods

### 3.1 Overview

To explore quantitative operationalizations of meaningful human control during dynamic task allocation in human-robot teams for firefighting, we conducted an online qualitative focus group. During the study, we presented several statements about and inspired by the operationalizations discussed in Section 2. In summary, we presented the following six statements: 1) Operationalizing the tracing condition can only be done using subjective measures; 2) the cascade approach evaluates all tracing aspects; 3) thresholding the final score of the cascade approach to determine sufficient tracing would be a good idea; 4) misses resulting from operator unawareness of moral sensitivity indicate the robot is not under meaningful human control; 5) misses when the operator is overloaded but aware of moral sensitivity indicate the robot is still under meaningful human control; and 6) the hit rate is an important property for the robot to be under meaningful human control, while the true discovery rate is not. All statements were formulated somewhat provocatively in an attempt to elicit strong responses. We invited experts in the field and asked them to respond to our statements while engaging in a discussion with each other. Data was collected from one focus group study with the experts and analyzed using reflexive thematic analysis (Braun and Clarke, 2019).

### 3.2 Data collection

Since the topic of operationalizing meaningful human control is complex and involves technical terminology and concepts not easily understood by laymen (e.g., the tracking and tracing conditions), we recruited five experts in the field of meaningful human control. To capture and represent the multifaceted nature of the topic, we recruited experts with various backgrounds such as engineering 1), law 1), human factors 1), and computer science 2). All participants published articles on meaningful human control and four of them specifically on operationalizing meaningful human control. Therefore, we believed the recruitment of these experts to facilitate the in-depth discussions and critical analyses required to generate concrete ideas on operationalizing meaningful human control during dynamic task allocation. We informed the expert participants that we would present statements on operationalizations of meaningful human control during dynamic task allocation using variable autonomy in human-robot teams for firefighting. Moreover, we asked them to respond to our statements while engaging in a discussion with each other. The first three statements corresponded to quantitative operationalization of the tracing condition, whereas the last three centered around objectively quantifying meaningful human control based on team and robot outcomes. All participants signed an informed consent form before participating in the study, which was approved by the ethics committee of our institution (ID 2477). The online focus group lasted around one and a half hours and was automatically transcribed using Microsoft Teams. Afterwards, this transcript was checked and improved using a video recording of the study, which was destroyed after this data processing step.

### 3.3 Data analysis

Data was analyzed using reflexive thematic analysis (Braun and Clarke, 2019), a method for producing a coherent interpretation of the data, grounded in the data. This approach is centered around the researcher’s role in knowledge production and subjectivity rather than achieving consensus between coders. Reflexive thematic analysis involves familiarization with the data, generating codes, constructing themes, revising and defining themes, and producing the report of the analysis. We outline the process for the first five phases below, the last phase is reported as Section 4. We first familiarized ourselves with the data by fine-tuning the transcription of the focus group using the video recording. Next, we read the full transcript in detail to double check for potential mistakes during the transcription process. During this step we already highlighted and took notes of potentially interesting text excerpts.

We systematically coded the transcript by searching for instances of talk that produced snippets of meaning relevant to the topic of operationalizing meaningful human control. These instances were coded using comments in Microsoft Word, highlighting the relevant text excerpt for each code. The coding of thematic analysis can be either an inductive approach, deductive approach, or combination of the two. This decision depends on the extent to which the analysis is driven by the content of the data, and the extent to which theoretical perspectives drive the analysis. Coding can also be semantic, where codes capture explicit meaning close to participant language, or latent, where codes focus on a deeper, more implicit or conceptual level of meaning. We used a deductive coding approach driven by prior literature and existing operationalizations, as well as operationalization ideas that we formulated. Semantic codes capturing explicit meaning close to participant language were noted, such as “challenges the use of thresholds”.

During the construction, revision, and definition of our themes, we first sorted our codes into topic areas using bullet-point lists. Next, we used visual mapping (using Miro) and continuous engagement with the data to further construct, revise, and define our themes. These candidate themes were grouped into one overarching theme of quantitative operationalization of meaningful human control, which encompassed ten themes and eight sub-themes. The process of revising and defining themes again involved visual mapping and continuous engagement with the data, mainly to check for relationships between themes. For example, we checked whether initial themes should be sub-themes of other themes, or whether sub-themes could be promoted to themes. Finally, this resulted in our full thematic map of six themes and eleven sub-themes. We grouped these themes and sub-themes into the overarching theme “quantitative operationalization of meaningful human control during dynamic task allocation using variable autonomy in human-robot teams for firefighting”.

## 4 Results

**FIGURE 1:**
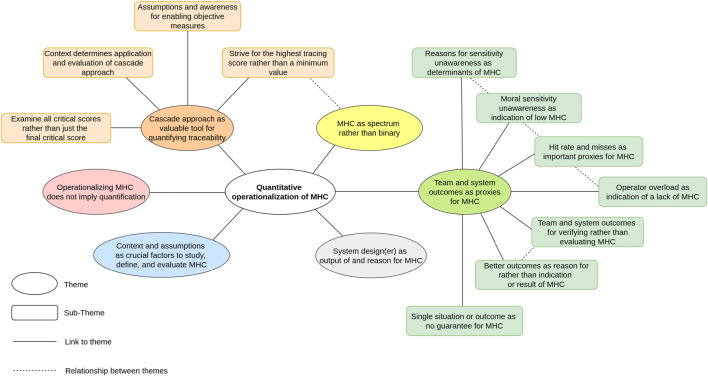
Thematic map on quantitative operationalization of meaningful human control (MHC) during dynamic task allocation using variable autonomy in human-robot teams for firefighting.

Our analysis revealed that the following six themes are underlying the main overarching theme: 1) The cascade approach as valuable tool for quantifying traceability; 2) meaningful human control as a spectrum rather than binary; 3) team and system outcomes as proxies for meaningful human control; 4) context and assumptions as crucial factors to study, define, and evaluate meaningful human control; 5) system design(er) as output and reason for meaningful human control; and 6) operationalizing meaningful human control does not imply quantification. Below we will discuss these six themes in detail. The full thematic map can be seen in Figure 1. Some themes consist of several sub-themes and were constructed based on extensive discussions clearly highlighting their importance (such as the theme described in Section 4.3). On the other hand, some themes do not consist of any sub-themes and were constructed based on briefer discussions that still highlighted significant relevance to be main themes (such as the theme described in Section 4.2). Although these themes are described in less detail, this does not mean they are less important.

### 4.1 The cascade approach to quantify traceability

The first main theme that we identified was “the cascade approach as valuable tool for quantifying traceability”. Most participants viewed the cascade approach as a valuable tool to quantify traceability during dynamic task allocation using variable autonomy. One participant considered the approach “as a first step to see how one could start to evaluate something which in itself is not quantifiable”. Most participants also shared how the approach is not perfect or the only method, but at the same time none of the experts were aware of (better) alternatives for quantitative evaluation of the tracing condition. Another expert explained how the cascade approach can also be used: “The cascade approach can give an indication, but then there should still be a human who can evaluate if the tracing condition is met based on the indication that the method gives.”

One sub-theme that we identified within this theme was “context as determining the application and evaluation of the cascade approach”. Some participants mentioned that the cascade evaluation approach misses some tracing aspects. Along those lines, most experts shared how context and level of abstraction are important for determining how to apply the approach and whether the approach evaluates all tracing aspects. For example, one expert explained: “I do think the cascade approach misses something, but I believe that if you make it more context specific you stand a chance at capturing the key aspects of tracing.”

Another sub-theme that we identified was “assumptions and awareness as enabling objective measures of operator understanding”. One expert speculated how the traceability aspect operator understanding of the robot can also be measured objectively, but that this requires assumptions about specific scenarios. This participant further mentioned the use of situation awareness and operational tests for measuring operator understanding objectively: “Let us assume a certain scenario for the operator. You could then test the operator to see if the operator is aware of what the robot might do in a certain circumstance. You can then let the robot perform the task and you can check to see if that is actually met. It is very hard to generalize this, but you can do this for very specific situations and then also objectively measure understanding in those specific situations.”

We also identified the sub-theme “examine all critical scores rather than just the final critical score”. This sub-theme is related to comparing the four traceability aspects of the cascade approach to determine critical scores, as discussed in Section 2.3. One of the experts explained: “If you accumulate the individual critical scores into one final score then you are removing information. So, it depends on the purpose of the tracing score, but I would be more interested in the individual scores that are composing the final score.” All the other participants agreed with this point. Another expert mentioned how considering both individual scores and final score can be relevant for comparing different robots, and that the individual scores can provide more information about which robot is easier to correct in order to improve traceability.

The final sub-theme that we constructed was “strive for the highest tracing score rather than a minimum value”. When discussing the use of a threshold to define when the final critical score reflects sufficient fulfillment of the tracing condition, all experts articulated how the goal should be to get the highest possible final score rather than a minimum value reflecting sufficient traceability. Furthermore, they mentioned how the critical scores are subjective and therefore it is inaccurate, not possible, and not necessary to set a threshold defining sufficient traceability. Finally, one expert explained how the scores of the cascade evaluation approach are more useful to inform rather than automate: “I would even challenge the very notion of thresholding because it reflects the kind of intrinsic desire to quantify everything. It does feel like this subjective cascade approach might actually inform decision-making without automating it because a threshold is a way of automating the decision.”

### 4.2 Meaningful human control as a spectrum

The second theme that we identified is closely linked to the previously discussed sub-theme. We called this theme “meaningful human control as a spectrum rather than binary”. Two participants explicitly mentioned that the evaluation of the presence of meaningful human control is more nuanced than saying yes or no and should be considered as a spectrum instead: “Meaningful human control itself as well as its different conditions is never black and white, is not binary, it is a spectrum basically. So there is an extent of meaningful human control, but it is never that there is full meaningful human control or there is zero.” The other experts all seemed to agree with this viewpoint.

### 4.3 Outcomes as proxies for meaningful human control

The third main theme that we constructed was “team and system outcomes as proxies for meaningful human control”. We identified this theme during the discussion of using signal detection theory to classify operator responses during during dynamic task allocation using variable autonomy. One sub-theme that we identified was “better outcomes as reason for rather than indication or result of meaningful human control”. Most participants shared how the quality of team and robot outcomes is not always an indication or a result of meaningful human control. One expert explained: “A bad outcome is not always an indication of a lack of meaningful human control and a good outcome is not always an indication of meaningful human control being present. It could also be that the human who is in control has made an error.” Similarly, another participant complemented: “Meaningful human control also does not equate to moral acceptability of any situation. A system can be under meaningful human control and show very questionable outcomes.” On the other hand, two experts articulated that one of the reasons for pursuing meaningful human control is to achieve better and ethically sound outcomes.

Another sub-theme that we constructed was “team and system outcomes for verifying rather than evaluating meaningful human control”. One expert questioned the correctness of assessing meaningful human control in terms of team and robot outcomes. Another expert agreed that outcomes alone are not sufficient for determining the presence of meaningful human control, but explained that “you can use outcomes to see whether they are in accordance with guidelines.” Moreover, this expert mentioned how outcomes can be used to verify the presence of meaningful human control rather than evaluate it. The expert who initially raised questions agreed with these points: “I totally agree that there is a relationship between meaningful human control and outcomes. Maybe the outcomes can be indirect evidence of meaningful human control.”

We also identified the sub-theme “single outcome or situation as no guarantee for meaningful human control”. Two participants mentioned how considering single situations and outcomes is not sufficient for determining the presence of meaningful human control: “A bad outcome is not always an indication of a lack of meaningful human control and a good outcome is not always an indication of meaningful human control being present. If you have enough situations and samples, then it does give a very good overall picture, but for one specific situation it does not give that guarantee. Sometimes in isolation a situation can be a bit misleading.”

Another constructed sub-theme was “hit rate and misses as important proxies for meaningful human control”. In addition to classifying operator responses during dynamic task allocation as hits, misses, false alarms, and correct rejections, we explained participants the distinction between the hit and true discovery rate. More specifically, the hit rate refers to the percentage of relevant situations where the operator correctly intervenes (by dividing hits by hits and misses). In contrast, the true discovery rate refers to the percentage of operator interventions which are necessary (by dividing hits by hits and false alarms). Two experts articulated how the true discovery rate and false alarms are not so important in relation to meaningful human control, while the hit rate and misses are: “If you intervene in the sense that you keep awareness and keep responsibility to yourself, even though there was not a necessary situation, I would say that the true discovery rate is not an important property for a system to be under meaningful human control, whereas the hit rate is.” Similarly, the other expert mentioned: “As long as the operator intervenes it does not really matter if they intervene even in the situation when the robot is kind of okay, but it does matter when the human does not intervene when the robot is not okay.”

We also identified the sub-theme “operator overload as an indication of a lack of meaningful human control”. All participants felt that misses resulting from operator overload indicate a lack of meaningful human control. The experts explained several reasons, such as “the system is in operation outside of what is reasonable to expect for that person”, “you need the ability to intervene in time and in a proper fashion”, and “this is an example of operator capacity being lower than their responsibility.”

Another sub-theme that we constructed was “moral sensitivity unawareness as an indication of low meaningful human control”. Most participants shared that misses resulting from operator unawareness of the moral sensitivity indicate low meaningful human control. One expert explained: “If the robot misinterprets the situation but the operator does not intervene, then there is obviously a lower level of meaningful human control because the robot in its design has not been able to identify the situation correctly and also the operator does not correctly intervene”. Another expert elaborated on the distinction between human control and meaningful human control: “Strictly speaking the system is under control of the human operator because he/she has the capability and the authority to intervene. So, strictly speaking, I should say it is under control of the human operator, but that does not necessarily imply meaningful human control.”

The final sub-theme that we constructed was “reasons for moral sensitivity unawareness as determinants of meaningful human control”. All participants mentioned how knowing the reasons for the operator’s unawareness of the moral sensitivity is very important for determining the extent of meaningful human control. For example, one expert mentioned how there would be no meaningful human control if the operator does not have the means to be aware of the moral sensitivity. Another participant explained: “Is the operator unaware because he/she cannot do anything about it, then the operator should not be held responsible. Then, the question is, depending on how the system was designed, does this lead to a responsibility gap or does this mean that responsibility should be attributed to a designer or someone else?”.

### 4.4 Context and assumptions as crucial factors

Another theme that we identified during the discussion of the previously reported sub-theme was “context and assumptions as crucial factors to study, define, and evaluate meaningful human control”. Two participants mentioned the importance of communicating the assumptions of our definitions, variable autonomy approach, and robot design and communication. For example, one expert explained: “I think what is very important when you do this, because I see the value, is communicating the assumptions you are using. Basically, you want to create a shared mental model.”

### 4.5 Design(er) and meaningful human control

We also identified the theme “system design(er) as output of and reason for meaningful human control”. One expert shared how operationalizing meaningful human control can result in requirements for robot design: “It does feel to me that one of the major benefits of operationalizing meaningful human control through tracking and tracing is to arrive in every individual context at a set of very context specific requirements for the design of the system”. On the other hand, several participants mentioned how meaningful human control can be present by system design and how both the system designer and robot operator should be considered during the discussion. For example, one expert shared: “You basically have two human agents here, you have the operator and you have the robot designer. If at least one of them is able to influence the situation in a way that human control is meaningful, then meaningful human control is still present. This does not have to be the operator necessarily, it can also be the way the robot is designed.”

### 4.6 Qualitative operationalization of meaningful human control

The final theme that we identified during the focus group study was “operationalizing meaningful human control does not imply quantification”. Most experts mentioned that quantification of meaningful human control has its value, but that it is very difficult to accurately quantify its elements and conditions. Moreover, two participants shared that operationalizing meaningful human control does not require or mean quantifying it. More specifically, one expert explained: “I just want to challenge the kind of implicit assumption here that operationalizing the tracing condition would require quantifying it, because I think that you can actually operationalize any notion to some extent in a completely qualitative way.”

## 5 Discussion and conclusion

### 5.1 Discussion

Our results emphasize the usefulness of the cascade approach to quantify traceability during dynamic task allocation using variable autonomy in human-robot teams for firefighting. Moreover, the results highlight a new application of the approach in comparing how different robot implementations or variable autonomy approaches affect traceability, for example, in terms of robot behavior, explanations, or autonomy adjustments. Another novel suggested application is, comparing all individual aspect scores as well as the final critical score to provide relevant information about traceability and potential points of improvement. These applications are novel compared to its original usage of evaluating implemented robots or variable autonomy approaches using only the final traceability score. The results further emphasize the importance of using a scale for such comparisons, where certain robot implementations or variable autonomy approaches may exhibit varying levels of traceability. Ultimately, the goal should be to get the highest possible traceability score rather than a minimum sufficient value. This is in line with some earlier interpretations of meaningful human control as ratio rather than binary (Calvert et al., 2020; Cavalcante Siebert et al., 2022). On the other hand, it contradicts the discussion on defining how much of each of the four properties for human-robot teams to be under meaningful human control is sufficient (Cavalcante Siebert et al., 2022).

**FIGURE 2:**
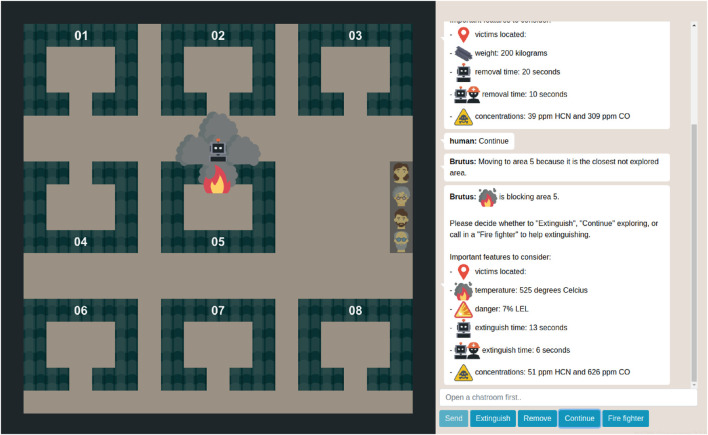
Simulation of the collaboration between the human operator and explore and extinguish robot during dynamic task allocation using variable autonomy. The robot is autonomously exploring an office building to search and rescue victims. The human operator is supervising the robot and they communicate via a chat box. When the robot perceives the need for a moral decision, it allocates decision-making to the human operator. All non-moral decisions are made by the robot.

Results also highlight a new application of situation awareness and operational tests to objectively measure the traceability aspect “human ability to understand and interact with a robot”. The (modified) Situation Awareness Global Assessment Technique (SAGAT) can be used to objectively measure human understanding of the robot during simulations of representative tasks (Sanneman and Shah, 2020; Verhagen et al., 2022). This way, SAGAT can also be used to investigate whether certain robot explanations can increase traceability by improving human understanding of the robot. Figure 2 shows an example of what a simulated task could look like for dynamic task allocation using variable autonomy in human-robot teams for firefighting. This simulated task is especially valuable for evaluating human-robot collaboration before real-world deployment, enabling easier manipulation and evaluation of aspects like robot communication and behavior (Van Zoelen et al., 2021; Schadd et al., 2022; Schoonderwoerd et al., 2022; Verhagen et al., 2022). For example, the simulated task in Figure 2 allows the evaluation of different robot explanations such as textual, visual, or hybrid explanations (Szymanski et al., 2021). Moreover, it allows the rapid implementation and evaluation of different variable autonomy and control approaches such as having a human-in-the-loop or having a human-on-the-loop.

The results further highlight the use of team and robot outcomes as verification of meaningful human control during dynamic task allocation using variable autonomy in human-robot teams for firefighting. In terms of outcomes, operator hits and misses during robot allocation of decisions are considered to be more important than false alarms and correct rejections. These objective outcomes provide novel measures for assessing the operator’s supervision performance during dynamic task allocation using variable autonomy. Using these team and robot outcomes to verify meaningful human control corresponds with the operationalization by van der Waa et al. (2021) that uses behavioral compliance with moral values and ethical guidelines as evidence for meaningful human control. Our results also emphasize that meaningful human control can be present by system design. This aligns with the claim that robots with variable autonomy can ensure meaningful human control over these robots (Methnani et al., 2021). It also aligns with our expectation that dynamic task allocation using variable autonomy in human-robot teams for firefighting can ensure meaningful human control by design. However, this could be verified using measures like the hit rate and whether outcomes are in accordance with firefighting guidelines, such as evacuating victims first when the location of the fire source is unknown and smoke spreads fast. Finally, the results introduce a new perspective by stressing the importance of collecting outcomes during multiple task simulations because single positive/negative outcomes are not always an indication of the presence/absence of meaningful human control.

Finally, our results highlight a novel perspective by emphasizing the importance of qualitatively identifying reasons underlying outcomes, such as why an operator is overloaded or unaware of moral sensitivity, to determine the extent of meaningful human control and how to increase it. Therefore, we believe that conducting follow-up interviews after completing simulations of representative tasks can be particularly effective to identify reasons underlying outcomes. For example, after the simulated task operators could be questioned about reasons for misses and how to improve the variable autonomy approach to avoid them. More specifically, if the operator has a low hit rate during the task, follow-up interviews could determine whether this results from an overload of robot information or unawareness of moral sensitivity due to limited experience. This distinction is crucial because these reasons determine the extent of meaningful human control and how to improve it. For example, an overload of robot information indicates a lack of meaningful human control requiring robot improvements such as decreasing robot communication. On the other hand, operator unawareness of moral sensitivity due to limited experience indicates low meaningful human control that can be addressed by more operator training.

In summary, the following novel knowledge on operationalizing meaningful human control has been gained because of the expert study. First, using the cascade approach for comparing different robot implementations or variable autonomy approaches, and not by comparing only the final critical score but also all individual aspect scores. Furthermore, using situation awareness and the hit rate to objectively measure the traceability aspect “human ability to understand and interact with the robot”, and collecting these measures during multiple task simulations for a more robust indication of meaningful human control. Finally, qualitatively identifying reasons underlying outcomes, like operator overload or moral unawareness, to determine the extent of meaningful human control and how to increase it.

### 5.2 Evaluating meaningful human control during dynamic task allocation

Based on these results, our main contribution is proposing the following evaluation method of meaningful human control during dynamic task allocation using variable autonomy in human-robot teams for firefighting. We suggest adapting the cascade approach to not only subjectively quantify traceability, but also objectively using operator responses during and after task simulations. The first aspect of the cascade approach involves scoring the exertion of operational control by human and robot separately, and the maximum of these scores is taken as the critical score of this aspect. This aspect can be scored a-priori based on the fixed collaboration characteristics during dynamic task allocation, where the robot exercises more operational control as it makes all non-moral decisions and handles allocation of decision-making. However, we suggest combining this a-priori score with an a-posteriori score determined by the involved human him/herself, for example, by measuring experienced control (van der Waa et al., 2021).

The second aspect of the cascade approach involves scoring the involvement of the human operator, and the minimum of this score and the critical score of the first aspect determines the critical score of the second aspect. The involvement of the human operator can also be scored a-priori based on the expectation of continuous supervisor involvement during dynamic task allocation. However, we suggest combining this a-priori score with an objective measure of situation awareness, which assesses the human operator’s perception, comprehension, and projection of environmental elements (Endsley, 1988). Here, higher situation awareness can be taken as a higher human operator involvement during the task. Situation awareness can be measured objectively using the traditional Situation Awareness Global Assessment Technique (SAGAT) (Endsley, 1988; Endsley, 2017). This involves a-priori defining the information and situation awareness requirements of the operator using goal-directed task analysis. Next, SAGAT queries should be formulated that objectively evaluate operator knowledge of this situational information. These queries should be asked during random pauses of the task simulation, either once or multiple times. The percentage of correctly answered queries can then be used as objective measure of situation awareness.

The third aspect of the cascade approach involves scoring the ability of the human operator to 1) understand the robot and 2) interact with the robot. The minimum of these two scores is then compared with the critical score of the second aspect, and the minimum of this comparison determines the critical score of the third aspect. We suggest to objectively measure the ability of the human operator to understand the robot using situation awareness of the robot’s behavior processes and decisions. In addition to measuring situation awareness, SAGAT is also suitable for objectively measuring human understanding of explainable systems like the robot dynamically allocating tasks (Endsley, 1988; Sanneman and Shah, 2020; Verhagen et al., 2022). In this case, the goal-directed task analysis involves the definition of situational information requirements specifically related to robot behavior. Again, the task simulation should be paused at random times, followed by evaluating operator knowledge of the predefined informational needs. Furthermore, we suggest to objectively measure the ability of the human operator to interact with the robot using task performance. Task performance can be determined by the operator’s hit and true discovery rates during the robot’s dynamic allocation of decision-making, where higher hit and true discovery rates would refer to better performance. Finally, the minimum score of the human ability to 1) understand the robot and 2) interact with the robot is taken as the score that is compared with the critical score of the second aspect.

The fourth and final aspect of the cascade approach involves scoring the ability of the human operator to understand their moral responsibility over the robot, and the minimum of this score and the critical score of the third aspect determines the final traceability score of the variable autonomy approach. We suggest quantifying this aspect using a semi-structured interview after completing the task simulation. This semi-structured interview can efficiently be followed by open questions to identify reasons underlying outcomes like operator misses. Identifying these reasons is crucial to further improve the variable autonomy approach, for example, by adjusting robot communication if many operators suffer from information overload. Finally, we suggest combining this subjective measure of moral responsibility understanding with an objective measure of how many outcomes adhere to ethical firefighting guidelines, such as not sending in firefighters when temperatures exceed auto-ignition temperatures of present substances. This way, not only the subjective understanding is considered but also translation of that understanding into adherence to ethical guidelines.

Our initial goal was a quantitative operationalization of meaningful human control during dynamic task allocation using variable autonomy in human-robot teams for firefighting. Ultimately, we propose a hybrid operationalization where some required qualitative elements (reasons underlying outcomes) supplement the quantitative elements (traceability aspects). During evaluation, we recommend using all aspects scores instead of just the critical scores to arrive at improvements for the variable autonomy approach. For example, consider an overloaded human operator with the critical aspect scores 3, 3, 0, and 0; and a inexperienced human operator with the critical aspect scores 3, 3, 2, and 2. Closer inspection of all aspect scores could reveal that the overloaded operator only lacks the ability to interact with the robot. Similarly, inspecting all scores of the inexperienced operator could reveal that the operator suffers from a low ability to both understand and interact with the robot and understand their moral responsibility over the robot. So, while the overloaded operator has a lower traceability score than the inexperienced operator, analyzing all scores suggests that the traceability score of the overloaded operator can be improved more easily as it results from only one aspect score instead of three.

### 5.3 Limitations

We identify a few limitations of our work. First of all, we conducted a single focus group that was coded individually. It can be favourable to conduct the same focus group multiple times with different experts, until reaching a saturation point. However, since our goal was to capture a particular perspective within a specialized domain, we considered one focus group appropriate to reach our objectives. Furthermore, thematic analysis is often associated with achieving consensus between multiple coders and high inter-coder reliability. However, it was our goal to generate rich, contextually situated, and nuanced themes instead. Therefore, we employed reflexive thematic analysis, emphasizing the researcher’s role in knowledge production and centering around researcher subjectivity (Braun and Clarke, 2019). All in all, while we acknowledge the limitations associated with a single focus group and individual coding, these were strategic choices aligned with our research objectives and the specialized nature of our domain.

Another limitation concerns the generalizability of our proposed evaluation method for meaningful human control (Section 5.2). Since this method is tailored to evaluating meaningful human control during dynamic task allocation using variable autonomy in human-robot teams for firefighting, it is questionable how it would translate to different contexts and systems. However, this is not necessarily a problem as the conditions, properties, and implementation of meaningful human control are context- and system-specific (Santoni de Sio and Van den Hoven, 2018; Cavalcante Siebert et al., 2022). On the other hand, we do believe some aspects can be used for different contexts and systems with similar levels of autonomy and outcomes. For example, objectively quantifying human ability to understand and interact with systems using situation awareness and task performance can also be done during simulations of drivers collaborating with automated driving systems. Here, even the hit and true discovery rates can be used when the task includes incorrect automated driving behavior requiring the human driver to intervene. Finally, we believe semi-structured interviews after task simulations can be generalized to all contexts and systems by providing a robust way to identify reasons underlying behavior and outcomes.

### 5.4 Future work

For future work, we want to verify if dynamic task allocation using variably autonomy indeed ensures meaningful human control in human-robot teams for firefighting. We believe that a user study in a simulated task environment similar to Figure 2 could provide valuable insights before considering in field tests. To implement the variable autonomy approach, the robot should be sufficiently able to identify morally sensitive situations (see Table 1). We are currently collaborating with the fire department of Rotterdam on this robot identification of morally sensitive situations. More specifically, we created a questionnaire to understand how people view morally sensitive situations in human-robot teams for firefighting. This questionnaire presents various situations during the collaboration between firefighters and their firefighting robot, such as locating the fire source, rescuing victims, and switching deployment tactic. These situations are characterized by different features such as the number of victims, fire duration, and fire resistance to collapse. In the questionnaire, participants specify how morally sensitive they consider each situation on a 7-point scale ranging from not morally sensitive to extremely morally sensitive (inspired by Reynolds (2006)). Moreover, they explain which feature(s) contributed the most to their rating and what feature changes would result in alternative moral sensitivity ratings. This way, we can identify which of the features are moral features and use them as predictors to statistically significantly predict the moral sensitivity of situations. This regression model can be implemented in the firefighting robot, together with a threshold for determining when the predicted moral sensitivity is too high and thus requires human decision-making. For future work, we want to first implement the dynamic task allocation and above mentioned regression model in a virtual robot and simulated environment similar to Figure 2. Next, we want to verify if dynamic task allocation indeed ensures meaningful human control during the collaboration.

To verify this, we need to measure meaningful human control during the user study. The results of our expert study will influence the measurement of meaningful human control during this user study in several ways, in line with our proposed evaluation method in Section 5.2. More specifically, we will determine the participants’ exertion of operational control a-priori based on the fixed collaboration characteristics during dynamic task allocation. The involvement of the participants as supervisors and understanding of the robot’s behavior will be determined by objective measures of situation awareness obtained by queries asked during random pauses of the task (Endsley, 1988; Endsley, 2017; Verhagen et al., 2022). We will determine the participants’ ability to interact with the robot using task performance, more specifically their hit rate during the robot’s allocation of moral decisions (i.e., do they intervene when the robot classifies morally sensitive situations as not morally sensitive). Finally, participants’ understanding of their moral responsibility over the robot will be measured after task completion, using a semi-structured interview. This interview will also be used to identify reasons underlying task performance.

In addition to verifying if dynamic task allocation ensures meaningful human control, we are particularly interested in which robot explanations can support the human operator to intervene and reallocate moral decision-making when the robot incorrectly classifies morally sensitive situations. To support the human operator during moral supervision of dynamic task allocation by the robot, robot explanations are crucial. For example, the robot can provide reason explanations underlying allocations (Baum et al., 2022), or explain the likely positive and negative consequences of decision options (Steen et al., 2022). The ultimate goal of these explanations is to raise human moral awareness by fulfilling the epistemic condition of direct moral responsibility (Rudy-Hiller, 2018; Baum et al., 2022). However, it is crucial that the robot explanations do not influence the human operator to hold the robot accountable (Lima et al., 2022). Instead, the robot explanations should make operators aware that robot behavior can be traced back to them and therefore they are in control and responsible for the outcomes (Veluwenkamp, 2022).

A final suggestion for future work is evaluating the consistency and generalizability of our proposed evaluation method of meaningful human control to different contexts and systems. It would be especially interesting to investigate how the method generalizes to variable autonomy approaches with higher levels of autonomy, for example, a completely autonomous artificial moral agent supervised by a human operator. Ultimately, these insights can result in a more general evaluation method of meaningful human control in human-robot teams using variable autonomy. Since designers of variable autonomy approaches lack metrics for systematically addressing meaningful human control while at the same time it is increasingly imposed as a requirement, such a general evaluation method would greatly benefit the field. All in all, our suggestions for future work can contribute to the further development of our evaluation method for meaningful human control and variable autonomy approach for human-robot firefighting teams.

### 5.5 Conclusion

In this study, we conducted a qualitative focus group on operationalizing meaningful human control during dynamic task allocation using variable autonomy in human-robot teams for firefighting, aimed at creating an evaluation method of meaningful human control for this scenario. Our results highlight the usefulness of quantifying the traceability condition of meaningful human control, especially for comparing different robot implementations or variable autonomy approaches. Furthermore, our findings suggest the use of objective situation awareness and performance to measure human ability to understand and interact with the robot. Results also highlight the use of team and robot outcomes to verify meaningful human control and the importance of identifying reasons underlying outcomes to improve the variable autonomy approach and determine the exact level of meaningful human control. Based on these results, we propose an evaluation method of meaningful human control during dynamic task allocation using variable autonomy in human-robot teams for firefighting. This method involves subjectively and objectively quantifying traceability using human responses during and after simulations of the collaboration. Moreover, the method involves semi-structured interviews after the simulation to identify reasons underlying outcomes and suggestions to improve the variable autonomy approach. Designers of variable autonomy approaches currently lack metrics to systematically address meaningful human control while at the same time it is increasingly imposed as a requirement of their approaches. Our evaluation method provides an important contribution that can verify if dynamic task allocation using variable autonomy in human-robot teams for firefighting ensures meaningful human control over the robot.

## Data Availability

The raw data supporting the conclusion of this article will be made available by the authors, without undue reservation.
